# Development, Implementation, and Evaluation of a Faculty Development Workshop to Enhance Debriefing Skills Among Novice Facilitators

**DOI:** 10.7759/cureus.6942

**Published:** 2020-02-10

**Authors:** Kamal Abulebda, Sushant Srinivasan, Tensing Maa, Anne Stormorken, Corrie E Chumpitazi

**Affiliations:** 1 Pediatric Critical Care Medicine, Riley Hospital for Children at Indiana University Health, Indianapolis, USA; 2 Pediatrics, University of Wisconsin School of Medicine and Public Health, Madison, USA; 3 Pediatrics, Nationwide Children's Hospital, Ohio State University College of Medicine, Columbus, USA; 4 Pediatrics, Rainbow Babies and Children's Hospital, Case Western Reserve University School of Medicine, Cleveland, USA; 5 Pediatrics/Emergency Medicine, Baylor College of Medicine, Houston, USA

**Keywords:** post-simulation debriefing, debriefing training, faculty development, novice debriefers

## Abstract

Introduction

Effective debriefing during simulation-based training (SBT) is critical to promote learning outcomes. Despite debriefing’s central role in learning and various published debriefing methods and techniques, little is known about faculty development structure for debriefing training among novice facilitators. Continuing medical education courses often use simulation-based methods but provide minimal training in debriefing techniques to novice facilitators. We describe the development, implementation, and evaluation of a structured debriefing training workshop for novice facilitators.

Methods

Designed and conducted by simulation debriefing experts, a debriefing workshop was provided to novice facilitators serving as faculty during the simulation-based Sedation Provider Course (PC) at the 2018 Society of Pediatric Sedation conference. Emphasizing evidence-based key elements of effective debriefing, the workshop was divided into three components: 1) an introductory 30 minute didactic, 2) 75 minutes role modeling of simulated effective and ineffective debriefing 3) 120 minutes repetitive deliberate practice sessions with summative and formative feedback. Effective transfer of learned debriefing skills was assessed during facilitators’ PC debriefing using the Objective Structured Assessment of Debriefing (OSAD) tool, facilitators’ self-efficacy, and PC student learners’ evaluation of facilitator debriefings during the PC.

Results

Sixteen facilitators participated in the 4-h workshop and the next day served as PC faculty. The median OSAD score was 31 (13-40) for all facilitators. OSAD components with lowest and highest performance were “Establishing Learning Environment” with a median score of 1 (1-5) and “Engagement of Learners,” with a median score of 4.75 (2.5-5). Facilitators' self- assessment in debriefing significantly improved on the 5-point Likert scale pre- and post-workshop, respectively. PC student learners’ evaluations revealed high degrees of satisfaction with debriefing quality.

Conclusions

A proposed model integrating full-length debriefing and repetitive practice paired with summative and formative feedback provides a feasible and effective approach for debriefing training of novice facilitators for simulation-based educational courses.

## Introduction

Simulation-based training (SBT) in healthcare is emerging as an important methodology for knowledge translation and facilitating acquisition of technical, non-technical skills, and behavioral skills [1-3]. SBT has been utilized by a wide range of clinical disciplines to provide a safe learning environment without exposing patients to preventable harm [4]. The use of simulation in healthcare has been shown to improve the performance of a healthcare provider in simulated and clinical settings, healthcare processes, and eventually patient outcomes [5-7]. For SBT to be effective, skilled facilitators need to guide learners during simulation training to optimize learning outcomes. This form of facilitated learning is termed debriefing, which is defined as a ‘discussion of actions and thought processes after an event to promote reflective learning and improved clinical performance [8-9]. Ultimately, this makes debriefing a key tenet of the experiential learning theory [10-12].

A large body of literature describes the process of effective debriefing models and methods of debriefing for SBT [13-15]. Essential elements include creating a safe learning environment, establishing a shared mental model, addressing key learning objectives, eliciting learners’ reactions, and maintaining an engaging environment for all learners in the debriefing process. Other studies have described the best assessment tools of effective debriefing following simulation events [16]. However, little is known regarding optimal teaching of debriefing skills to facilitators, notably type of knowledge translation, and duration or need for repetition and reinforcement of these skills over time. This is important, as facilitator training is recognized as an essential component to ensure maximizing learning opportunities arising from simulation events by identifying learning needs among learners and translating lessons learned to improve future clinical practices [17]. Additionally, debriefing quality and impact on learner outcomes are highly dependent on the performance of the debriefing facilitator. 

Historically, formal debriefing training has been provided through a variety of mechanisms. Pursuing simulation fellowship training or advanced degrees in simulation poses time and financial challenges to clinical educators who wish to combine this training with existing clinical and academic obligations. Other options include attending simulation instructor training courses, conference-associated workshops, and on-line modules [18-19]. Simulation instructor training courses are commonly single day or multi-day courses and are rarely focused solely on debriefing training, often including other learning objectives such as administering simulation programs or developing scenarios. Additionally, these courses offer limited opportunities to practice debriefing skills with peers and/or actors.

Curricular elements promoting effective debriefing skill acquisition and subsequent translation to debriefing practice are not well defined. Dedicated debriefing workshops should provide essential knowledge, debriefing practice opportunities and real-time feedback of acquired debriefing skills to achieve competency. Despite the prevalence of these workshops and courses, little has been published about the effectiveness of these opportunities in enhancing the debriefing quality and transfer of debriefing skills within simulation programs [20]. The most available evidence is subjective of self-reported assessments of instructors’ comfort in conducting debriefing following these courses [21]. Therefore, accurate assessment of debriefing skill acquisition during educational sessions is vital in evaluating the impact of debriefing training strategies [22].

In this article, we describe the development, implementation, and evaluation of a debriefing training workshop for novice facilitators. We hypothesized that our instructional design of the workshop would provide a professionally diverse faculty with the essential skills to provide effective debriefing based on the assessment of transfer of skills to debriefing practice.

## Materials and methods

Workshop theoretical framework

**Figure 1:**
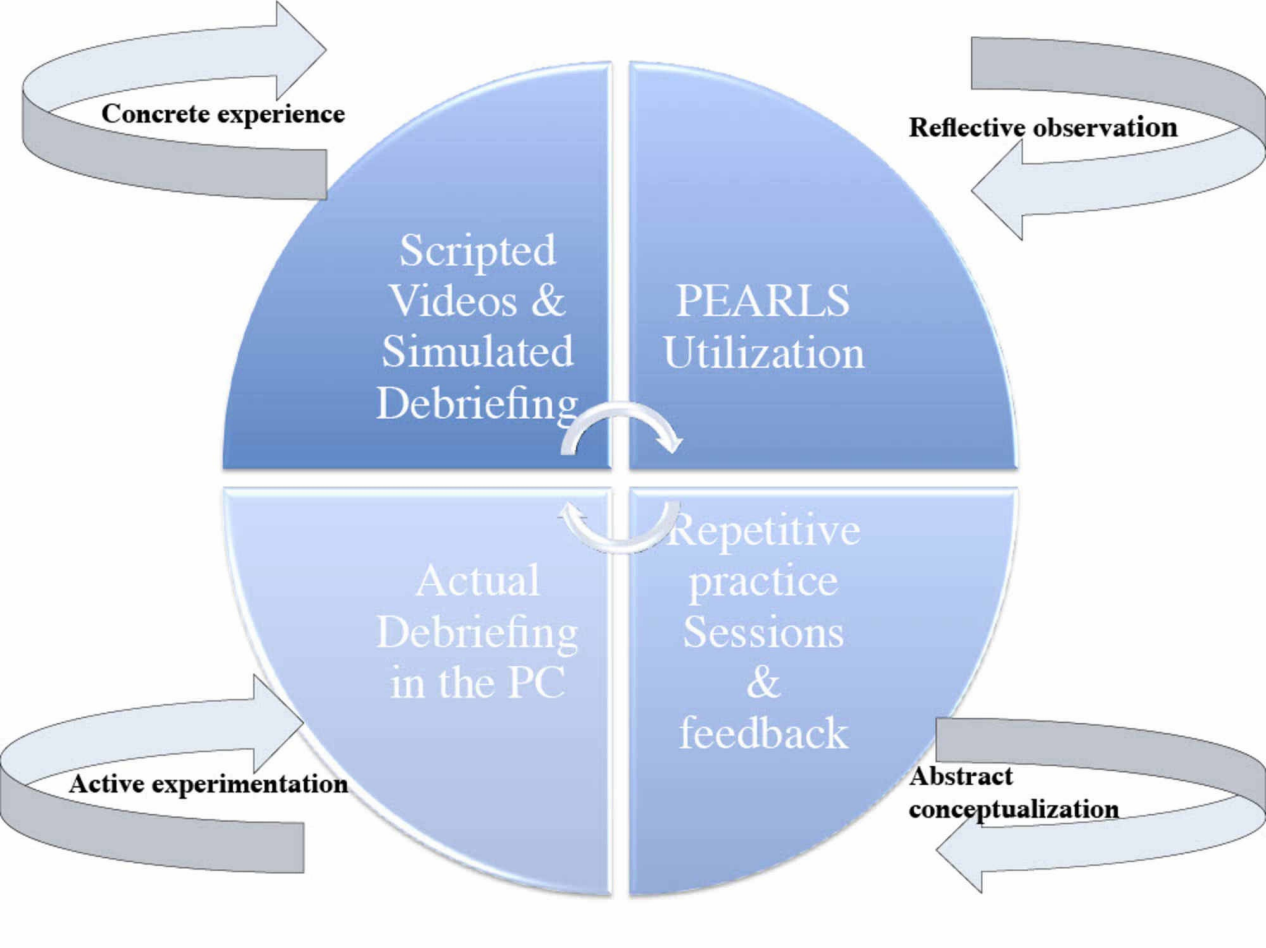
The theoretical framework of the debriefing training workshop

The theoretical frame of this workshop was based on using debriefing in fostering learning and behavioral changes based on Kolb’s theory of experiential learning [12]. In this theory, effective learning is observed when the learner progresses sequentially through these four stages: (1) having a concrete experience followed by (2) observation of and reflection on that experience which leads to (3) the formation of abstract concepts and generalizations which are then (4) used to test hypothesis in future situations, resulting in subsequent new concrete experiences. In SBT, the simulation event serves as the concrete experience of Kolb’s experiential learning cycle. The debriefing immediately following typically provides the chance for the learner to undergo reflection and conceptualization related to this ‘experience’. Eventually, learners will experiment with the new experience in future situations (Figure 1).

Needs assessment

Since 2011, the Society for Pediatric Sedation (SPS) has held annual Provider Courses (PCs), consisting of a full day of interprofessional simulation-based instruction intended to provide sedation practitioners with the essential knowledge and core competencies to promote safe and effective procedural sedation [23]. The SPS, which is a volunteered medical society, has utilized facilitators who are experts in pediatric sedation, however, their simulation backgrounds and debriefing skills varied. Debriefing sessions occurred with an unstructured format that tended to focus on the instructor experience and thus provided an opportunity to more effectively promote learning through the creation of a debriefing workshop. A debriefing workshop was created at the request of the course directors to provide a brief 4-h educational session the day prior to the SPS course to train the participating facilitators. Based on previous learner feedback and personal observation from lead instructors, workshop goals and objectives emphasized key elements of effective debriefing to these facilitators (Table 1). This study was exempted by the Institutional Review Board.

**Table 1:** Workshop learning objectives

1. Describe key components of effective debriefing (Psychosocial safety, basic assumption, establishing debriefing rules, addressing learning objectives, sharing mental model, asking open-end questions and using silence)
2. Describe the essential phases of the debriefing process (three phases or multi-phases)
3. Characterize good or bad debriefing techniques when observing a debriefing
4. Utilize best practice debriefing techniques to conduct an effective debriefing

Debriefing workshop faculty

A working group of four content experts in pediatric procedural sedation and simulation debriefing (KA, CC, TM, SS) developed the workshop format and served as lead instructors. At the time of the debriefing workshop, all lead instructors were either actively leading or implementing SBT programs.

Participants

Sixteen facilitators were recruited from invited faculty for the 2018 SPS Course held in July in Atlanta, GA. These facilitators are content experts in pediatric sedation with diverse professional backgrounds including pediatric critical care, anesthesiology, emergency medicine, and hospital medicine. Table 2 presents the demographics and debriefing experience for the facilitators.

**Table 2:** Demographics and professional backgrounds of the facilitators Values are means (standard deviations); medians (ranges) for continuous variables and frequencies (percentages) for categorical variables. * Defined as running a simulation scenario or operating a simulator in the context of simulation-based learning or training ** Defined as attending/completing a formal one or multi-days simulation instructor course that is not dedicated or focused toward debriefing training ^ Defined as conducting post-simulation or post-clinical event debriefing based on individual skills with no formal training or a structured format of debriefing ^^ Defined as any formal or structured dedicated debriefing training format (workshop, boot camp, single/multiday course or fellowship) SPS: Society of Pediatric Sedation PC: Provider Course

Years of Practice	14 (2 – 24)
Sedation Experience	9 (0 – 20)
SPS PC Simulation Experience	3 (2 – 8)
Discipline N (%)	
Physician	14 (86.5%)
Nurse Practitioner	1 (6.25%)
Nurs	1 (6.25%)
Specialty N (%)	
Anesthesia	3 (18.7%)
General Pediatric	1 (6.2%)
Pediatric Hospital Medicine	5 (31.2%)
Pediatric Critical Care Medicine	5 (31.2%)
Pediatric Emergency Medicine	1 (6.2%)
Pediatric Sedation	1 (6.2%)
Simulation & Debriefing Experience N (%)	
Prior Simulation Experience/Exposure^*^	11 (68.75%)
Simulation Instructor Course Experience^**^	4 (25%)
Informal Debriefing Experience^^^	3 (18.75%)
Formal Debriefing Training or Course^^^^	0 (0%)

Curriculum design and educational strategies

Prior to the workshop, facilitators were encouraged to visit the debrief2learn.org website to familiarize themselves with a debriefing framework, “Promoting Excellence and Reflective Learning in Simulation” [24]. The lead instructors created two scripted videos of a sedation team simulating common adverse events: hemodynamic instability and upper airway obstruction with hypoxia. Three of the instructors (KA, CC, TM) acted as the sedation team and made obvious mistakes in technical skills, communication, and teamwork. The intentional mistakes provided opportunities for the facilitators to offer feedback during their debriefing practice sessions.

**Figure 2:**
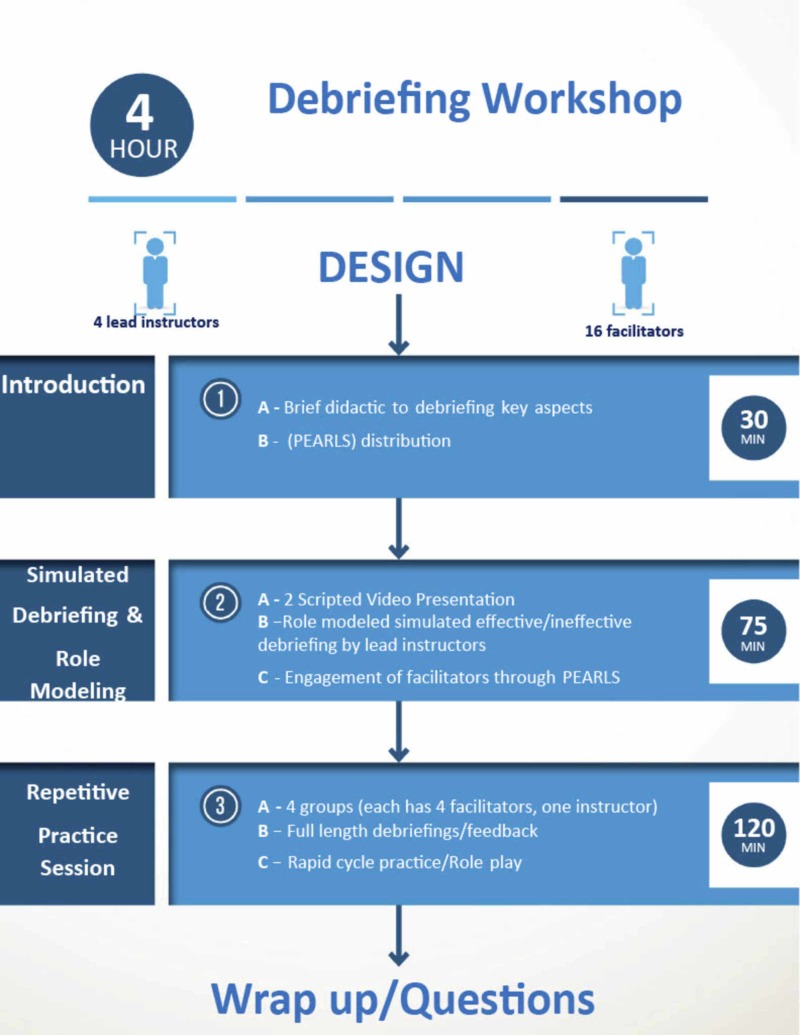
Workshop components

The workshop was divided into three components: 1) Introductory didactic presentation, 2) Simulated debriefing and role modeling of an ineffective and effective debrief, and 3) Repetitive deliberate practice sessions (Figure 2).

I- Introduction (30 min)

Introduction to the construct of debriefing, focusing on debriefing framework theory and key elements of effective debriefing. The PEARLS Healthcare Debriefing Tool was highlighted and facilitators used this tool as a reference throughout the workshop [13]. (Appendix 1)

II- Role Modeling of Effective and Ineffective Debriefing (75 min)

a) Ineffective debriefing: After showing all facilitators the hemodynamic instability video, the four lead instructors demonstrated a live ineffective debrief, wherein one instructor intentionally modeled an ineffective debriefing style while the remaining three instructors reprised their roles in the video. Using the PEARLS tool, facilitators were asked to reflect on their observations during the “simulated” debriefing and offer suggestions for improvement.

b) Effective debriefing: Following the ineffective live debriefing, the same instructor modeled an effective debriefing of the same scenario. During this live debrief, the instructors emphasized the essential components of effective debriefing, engaging the facilitators by utilizing the PEARLS tool as a guide. To conclude the session, the four lead instructors encouraged facilitators to summarize their noted observations related to the video scenarios and accompanying debriefings.

III- Repetitive Deliberate Practice Sessions (120 min)

This portion was dedicated to immersive simulation debriefing practice using the second video involving upper airway obstruction with hypoxia. Facilitators were divided into four groups, which were assigned to a lead instructor. Facilitators engaged in repetitive role-play, taking turns acting as the role of debriefer, while the others assumed the roles of sedation team members. All facilitators were provided the opportunity to function as the debriefer and receive formative feedback from their peers (debrief the debriefer). The lead instructor ensured that the PEARLS framework was utilized during these practice sessions as well as answer any questions. Each individual facilitator debriefing session lasted 25 min allowing for rapid cycles incorporating practice, feedback, and closed-loop learning. By the end of this 120-minute period, all facilitators had the opportunity to provide a full-length debriefing and participate in three other debriefings.

IV- Wrap-up Station (15 min)

Lead instructors reviewed the workshop learning objectives, discussed how these were met, and answered any facilitators’ questions. Self-assessment forms and workshop evaluation surveys were distributed to all facilitators.

Workshop evaluation

Debriefing skill acquisition during the workshop was evaluated during the Sedation PC the following day. The primary outcome of the workshop was to evaluate the effectiveness of facilitators’ debriefing sessions using the Objective Structured Assessment of Debriefing (OSAD) [25]. OSAD is a validated tool mirroring the core components of effective debriefing with good content validity to assess the effectiveness of debriefing following simulation and has been widely used as a tool to ensure a level of standardization in debriefing among individuals and institutions. OSAD has eight key aspects of debriefing each scored from one (minimum) to five (maximum). During the SPS PC, the faculty facilitators were required to primarily debrief twice without intervention by lead instructors. Each facilitator debriefing was assessed and scored by two lead instructors, one of which was real-time and the other through video review. Prior to the workshop, all lead instructors underwent rater training for the OSAD tool, viewing and scoring examples of effective and ineffective debriefings. Lead instructor interrater reliability was calculated and results included in (Table 3).

**Table 3:** Lead instructors interrater reliability

Reliability Statistics	
Cronbach's Alpha	N of Items
.980	4

Intraclass Correlation Coefficient							
	Intraclass Correlation^b^	95% Confidence Interval		F Test with True Value 0			
		Lower Bound	Upper Bound	Value	df1	df2	Sig
Single Measures	.905^a^	.756	.975	50.375	8	24	.000
Average Measures	.974	.925	.994	50.375	8	24	.000
Two-way random effects model where both people effects and measures effects are random.							
a. The estimator is the same, whether the interaction effect is present or not.							
b. Type A intraclass correlation coefficients using an absolute agreement definition.							

Reliability Statistics	
Cronbach's Alpha	N of Items
.992	4

Intraclass Correlation Coefficient							
	Intraclass Correlation^b^	95% Confidence Interval		F Test with True Value 0			
		Lower Bound	Upper Bound	Value	df1	df2	Sig
Single Measures	.958^a^	.879	.989	125.186	8	24	.000
Average Measures	.989	.967	.997	125.186	8	24	.000
Two-way random effects model where both people effects and measures effects are random.							
a. The estimator is the same, whether the interaction effect is present or not.							
b. Type A intraclass correlation coefficients using an absolute agreement definition.							

Secondary outcomes included facilitators’ assessment of their self-efficacy in debriefing using a questionnaire administered pre- and post-workshop. SPS PC learners’ evaluation of the facilitator debriefing skills was an additional secondary outcome.

Statistical analysis

Basic demographic and descriptive score information was generated and given as medians (ranges) for continuous variables and frequencies (percentages) for categorical variables. Changes in scores between pre- and post-workshop assessments were analyzed using a non-parametric signed-rank test to determine if the change was significantly different from zero (no change), due to data non-linearity. Interclass correlation analyses were performed using Generalized Estimating Equations models to account for the repeated measures of each facilitator. All analyses were performed using SAS v9.4 (SAS Institute, Cary, NC)

## Results

A total of 16 novice facilitators from pediatric subspecialties including critical care, emergency medicine, anesthesia, and hospital medicine participated in the debriefing workshop. The mean sedation experience for facilitators was 9.9 years. Eleven facilitators had prior simulation experience (69%) and four (25%) had formal simulation instructor training. Three (19%) facilitators had previous informal debriefing experience through participating in the previous PC while none of the facilitators had any prior formal debriefing training or experience. 

All 16 instructors conducted and debriefed two simulation sessions as faculty for the SPS PC following the workshop. Faculty assessment revealed that the median total OSAD score was 31 (13-40) with excellent inter-rater reliability (intra-class correlation coefficient 0.8248). Inter-rater reliability among the eight OSAD components was good to excellent (intra-class correlation coefficient range from 0.6850 to 0.9921). OSAD components with lowest and highest performance were “Establishing Learning Environment” with a median score of 1 (1-5) and “Engagement of Learners,” with a median score of 4.75 (2.5-5) with an intra-class correlation coefficient of 0.99 and 0.68, respectively (Table 4).

**Table 4:** Facilitators OSAD Scores and Faculty Intra-class Correlation Descriptive statistics are taken across both raters. ICC ranges from 0 to 1, with values closer to 1 indicating better agreement. Values between 0.75 and 1.00 are considered excellent; between 0.60 and 0.74 are good; between 0.40 and 0.59 are fair; less than 0.40 are poor. OSAD: Objective Structured Assessment of Debriefing

Question	Mean (standard deviation); median (range)	Intra-class Correlation Coefficients
Approach	5 (2.5 – 5)	.8037
Environment	1 (1 – 5)	.9921
Engagement	4.75 (2.5 – 5)	.6850
Reaction	4 (1.5 – 5)	.8093
Reflection	4 (2 – 5)	.7416
Analysis	4 (1 – 5)	.6956
Diagnosis	4 (1 – 5)	.7228
Application	4 (1 – 5)	.7789
Total (possible range 8 – 40)	31 (13 – 40)	

 

Facilitators' self-assessment in debriefing significantly improved across the five domains of the questionnaire using the Likert scale before and after the course (Table 5).

**Table 5:** Facilitators self-assessment pre and post the workshop Values are medians (ranges) with p-value for non-parametric signed rank test, with p<.05 indicating a significant difference from zero change.

	Pre	Post	Change	p-value
1. Identify components of an effective debriefing	3 (1 – 5)	5 (4 – 5)	2 (0 – 4)	.0002*
2. Describe the essential phases of the debriefing process	2 (1 – 5)	4 (4 – 5)	2 (0 – 4)	.0002*
3. Describe the job of the debriefer during the debriefing process	3 (1 – 5)	5 (4 – 5)	2 (0 – 4)	.0001*
4. Identify effective or ineffective debriefing techniques when observing	3 (1 – 5)	4 (4 – 5)	1 (0 – 4)	.0005*
5. Successfully utilize debriefing techniques to conduct an effective debriefing	2 (1 – 5)	4 (3 – 5)	2 (0 – 3)	.0001*

Evaluations from the PC student learners (n=24) revealed a high degree of satisfaction with educational quality: they found the debriefing environment safe and non-threatening, felt that a friendly non-judgmental atmosphere was maintained, were engaged in self-reflective discussions, and felt all team members were engaged (Table 6).

**Table 6:** Learners’ evaluation of the facilitators debriefings

Item	Score out of 5
1. The debriefing environment was safe and non-threatening	4.9
2. Debriefers maintained a friendly non-judgmental atmosphere	4.9
3. Debriefers actively involved all team members in the discussion	4.9
4. Debriefers successfully addressed learning objectives	4.8
5. Debriefers allowed for learners to engage in self-reflective discussions	4.9
6. Debriefers successfully highlighted "take home" messages by the end of the debriefing session	4.8

## Discussion

Debriefing by skilled facilitators is a critical component of SBT as a key determinant of effectiveness [9, 11, 15]. The overall educational impact of debriefing is highly dependent on skilled faculty; hence formal faculty debriefing training is crucial. However, despite several proposed debriefing training methods, demonstration of debriefing skill acquisition has not been shown, and the ideal training format has yet to be determined [18-19].

In this study, we describe our experience in developing, implementing, and evaluating an educational model for facilitator debriefing training. Our workshop was successful in providing novice facilitators with essential skills to provide effective debriefing through assessment of their debriefing performances using the OSAD scoring tool. This was further emphasized by facilitator self-assessment surveys and learner evaluations of the debriefing experience. To our knowledge, this is the first study to integrate debriefing-specific training with an existing SBT course and use a validated tool to assess the impact on course faculty and learners. This pre-course debriefing workshop model could be incorporated into established simulation-based continuing medical education (CME) courses to provide faculty development in a convenient and minimally disruptive manner.

Our instructional design innovatively incorporated the use of many educational strategies in a blended learning system including didactic presentations, simulated debriefing by lead instructors, deliberate practice sessions, immediate feedback, and full-length debriefings. 

By using videos of simulated scenarios scripted to incorporate mistakes, workshop lead instructors were able to conduct live effective and ineffective debriefing sessions following the brief 30 minutes didactic session on debriefing theory. Demonstrating these concepts from the didactic learning with the use of the PEARLS debriefing tool during the live simulated debriefing, provided facilitators with an experiential event followed by immediate feedback to enhance learning. The live debriefing created an environment of trust to facilitate peer learning and reflection prior to the next station. This is particularly important, as some senior facilitators could have found it more challenging to accept feedback from their relative junior instructors. In creating this safe environment, we ensured enhanced feedback credibility allowing facilitators to be more “receptive” of feedback provided by the lead instructors throughout the next station.

A second innovative feature of this workshop was allowing each facilitator group to conduct a full-length debriefing as well as participate in three full debriefings of peer facilitators, concluding with constructive formative feedback by a lead instructor. This provided learners with experiential opportunities to apply learning from both didactic and video sessions thereby accelerating debriefing skill acquisition. This approach has been shown to be effective in other educational contexts [20, 26-27]. Furthermore, coupling full-length debriefing with formative feedback and repetitive role-play amongst facilitators has been proposed as a model for faculty debriefing training [20].

Given the adaptability of the PEARL tool to various learner groups, we have implemented its use for all facilitators during future debriefing training workshops and SPS PCs [24]. Since our facilitators have no previous formal training in debriefing, this approach provides structure and guidance for educating novice debriefers. This blended debriefing framework that has explicit debriefing steps and with possible wording choices provides an easy-to-implement debriefing tool to utilize during our faculty development workshop.

Following the workshop, facilitators’ debriefing performance was tracked during the PC through summative assessment using the OSAD tool providing quantitative measures of debriefing skills with potential use to track facilitators’ skills in future workshops. Using the OSAD assessment tool, the median overall facilitator score was 31 (13-40) out of a maximum possible score of 40, demonstrating workshop efficacy in providing clinicians from diverse professional backgrounds with the essential elements of effective debriefing. The majority of the OSAD dimensions (7 of 8) had an average score of 4/5 with only one dimension “establishing a safe learning environment” having an average score of 1/5. This could be attributed to the early introduction of this concept at the beginning of the PC and decreased emphasis on this component “establishing a safe learning environment” during subsequent debriefing sessions. Furthermore, workshop efficacy was demonstrated by the significant increase in facilitators’ self-assessment of debriefing skills post-workshop. Additionally, learners’ evaluation of the facilitators reflected high satisfaction with the essential components of the facilitators’ debriefing.

This study of debriefing workshop effectiveness has some limitations to consider. First, we did not assess facilitators’ debriefing skills prior to the workshop using the OSAD tool to compare to the improvement noted afterward given practical challenges of facilitator availability. A second limitation is the lack of learner evaluation of the individual facilitator’s debriefing; instead, we have learners’ evaluation of the overall debriefing experience, as evaluating individual facilitators would not have been feasible given the PC logistics as well as being subject to recall bias. Third, we have demonstrated only the immediate impact of the debriefing workshop on the transfer of skills to the PC the following day, which could have been confounded by the Hawthorne effect; therefore, future studies should investigate the long-term retention of gained debriefing skills over time. Fourth, two of the four instructors who scored the debriefings were not blinded to the facilitators' identity which could have introduced scoring bias, however, that was less likely as we demonstrated good to excellent inter-rater reliability among instructors.

In summary, maximizing learning outcomes of SBT requires the acquisition of effective debriefing skills by simulation facilitators. Given the paucity of evidence regarding optimal debriefing training format, we created a national meeting-based debriefing training workshop to teach effective debriefing to novice facilitators. We believe that our proposed model integrating full-length debriefing and repetitive practice paired with summative and formative feedback provides a feasible and effective approach to faculty training in debriefing for other simulation-based CME courses.

## Conclusions

Our innovative model integrating full-length debriefing and repetitive practice paired with summative and formative feedback provides a feasible and effective approach to faculty training. This model could be utilized for faculty training in debriefing for other simulation-based courses.

## Figures and Tables

**Figure 3:**
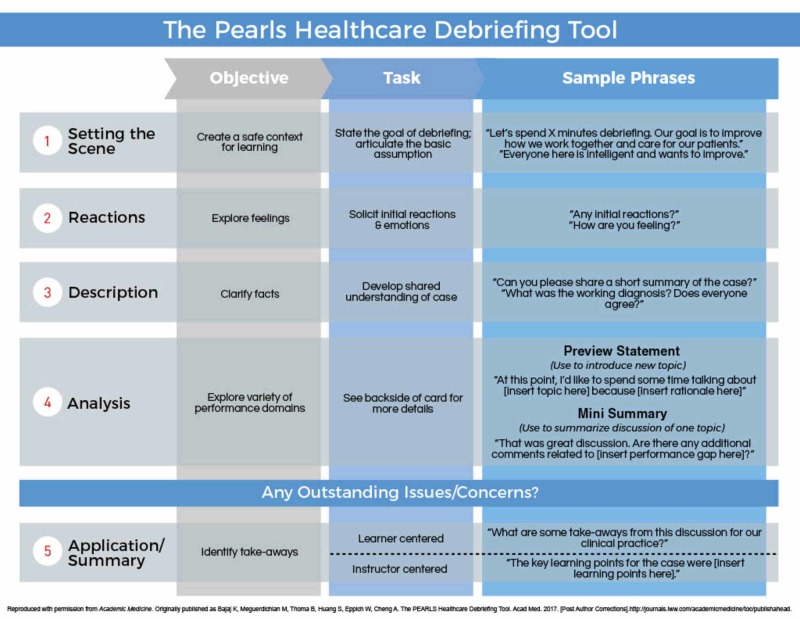
Supplemental 1: The PEARLS Tool

## Appendices

Appendix 1: The PEARLS Tool 
